# Adverse events during endovascular treatment of ruptured aneurysms: A prospective nationwide study on subarachnoid hemorrhage in Sweden

**DOI:** 10.1016/j.bas.2023.102708

**Published:** 2023-11-10

**Authors:** Bryndís Baldvinsdóttir, Paula Klurfan, Johanna Eneling, Elisabeth Ronne-Engström, Per Enblad, Peter Lindvall, Helena Aineskog, Steen Friðriksson, Mikael Svensson, Peter Alpkvist, Jan Hillman, Erik Kronvall, Ola G. Nilsson

**Affiliations:** 1 Department of Clinical Sciences, Neurosurgery, Lund University, Lund, Sweden; 2 Department of Clinical Neuroscience, University of Gothenburg, Gothenburg, Sweden; 3 Department of Clinical Sciences, Linköping University, Linköping, Sweden; 4 Department of Neuroscience, Section of Neurosurgery, Uppsala University, Uppsala, Sweden; 5 Department of Clinical Sciences, Umeå University, Umeå, Sweden; 6 Department of Clinical Neuroscience, Karolinska Institutet, Stockholm, Sweden

**Keywords:** Aneurysm, Subarachnoid hemorrhage, Endovascular, Adverse event, Complication, Outcome

## Abstract

**Introduction:**

A range of adverse events (AEs) may occur in patients with subarachnoid hemorrhage (SAH). Endovascular treatment is commonly used to prevent aneurysm re-rupture.

**Research question:**

The aim of this study was to identify AEs related to endovascular treatment, analyze risk factors for AEs and how AEs affect patient outcome.

**Material and methods:**

Patients with aneurysmal SAH admitted to all neurosurgical centers in Sweden during a 3.5-year period (2014–2018) were prospectively registered. AEs related to endovascular aneurysm treatment were thromboembolic events, aneurysm re-rupture, vessel dissection and puncture site hematoma. Potential risk factors for the AEs were analyzed using multivariate logistic regression. Functional outcome was assessed at one year using the extended Glasgow outcome scale.

**Results:**

In total, 1037 patients were treated for ruptured aneurysms. Of which, 715 patients were treated with endovascular occlusion. There were 115 AEs reported in 113 patients (16%). Thromboembolic events were noted in 78 patients (11%). Aneurysm re-rupture occurred in 28 (4%), vessel dissection in 4 (0.6%) and puncture site hematoma in 5 (0.7%). Blister type aneurysm, aneurysm smaller than 5 mm and endovascular techniques other than coiling were risk factors for treatment-related AEs. At follow-up, 230 (32%) of the patients had unfavorable outcome. Patients suffering intraprocedural aneurysm re-rupture were more likely to have unfavorable outcome (OR 6.9, 95% CI 2.3–20.9).

**Discussion and conclusion:**

Adverse events related to endovascular occlusion of a ruptured aneurysm were seen in 16% of patients. Aneurysm re-rupture during endovascular treatment was associated with increased risk of unfavorable functional outcome.

## Highlights

• Aneurysmal subarachnoid hemorrhage is a severe type of brain bleeding. Adverse events related to occlusion of the aneurysm can occur and may have effect on patient outcome.
• Occlusion of the aneurysm is commonly performed with an endovascular approach.
• A prospective nationwide study on adverse events related to the endovascular treatment, risk factors for these events and their impact on outcome has not been published before.
• Intraprocedural thromboembolic events and aneurysm re-rupture were seen in 11% and 4% of the treated patients, respectively.
• The presence of risk factors for adverse events in patients with subarachnoid hemorrhage may be of relevance in selecting the occlusion method of a ruptured intracerebral aneurysm.

## Contributorship statement

Bryndís Baldvinsdóttir MD: Data collection, Data analysis and interpretation, Drafting the article, Critical revision of the article, Approval of the submitted article, Corresponding author, Co-writer of the cover letter, Paula Klurfan MD: Data collection, Critical revision of the article, Approval of the submitted article, Johanna Eneling MD: Data collection, Critical revision of the article, Approval of the submitted article, Elisabeth Ronne Engström MD PhD: Designing the study database, Data collection, Critical revision of the article, Approval of the submitted article, Per Enblad MD PhD: Designing the study database, Data collection, Critical revision of the article, Approval of the submitted article, Peter Lindvall MD PhD: Designing the study database, Data collection, Critical revision of the article, Approval of the submitted article, Helena Aineskog MD: Data collection, Critical revision of the article, Approval of the submitted article, Steen Friðriksson MD PhD: Designing the study database, Critical revision of the article, Approval of the submitted article, Mikael Svensson MD PhD: Designing the study database, Data collection, Critical revision of the article, Approval of the submitted article, Peter Alpkvist MD: Data collection, Critical revision of the article, Approval of the submitted article, Jan Hillman MD PhD: Designing the study database, Data collection, Critical revision of the article, Approval of the submitted article, Erik Kronvall MD PhD: Data collection, Data analysis and interpretation, Drafting the article, Critical revision of the article, Approval of the submitted article, Ola G. Nilsson MD PhD: Designing the study database, Data collection, Data analysis and interpretation, Drafting the article, Critical revision of the article, Approval of the submitted article, Co-writer of the cover letter.

## 2 Introduction

Despite substantial improvements in the management of aneurysmal subarachnoid hemorrhage (SAH) during the last decades, morbidity and mortality remain high (Nieuwkamp et al., 2009; Huhtakangas et al., 2015) The impact of the initial bleed, secondary insults during the ensuing clinical course and adverse events (AEs) related to treatment affect functional outcome (Al-Khindi et al., 2010; Wong et al., 2012a; Wong et al., 2012b; Baldvinsdóttir et al; Chen et al., 2014). Occlusion of the aneurysm can either be done by microsurgery or endovascular technique, both of which carry certain benefits and risks.

Coiling is most often the endovascular treatment of choice for ruptured aneurysms. Other endovascular treatment methods are stent-assisted coiling, placement of a flow diverter, a Woven EndoBridge (WEB) device, or parent artery occlusion (Pierot and Wakhloo, 2013). For the past two decades, notably after the publication of ISAT (Molyneux et al., 2005), endovascular treatment has been the method of choice for most types of ruptured aneurysms in many centers (Currie et al., 2011).

Complications and/or AEs are not uncommon in the treatment of SAH (Baldvinsdóttir et al.; Chen et al., 2014; Pierot et al., 2010). Well-known intraprocedural AEs related to endovascular aneurysm treatment are thromboembolism, aneurysmal re-rupture, vessel dissection and hematoma at the puncture site (Currie et al., 2011; Pierot et al., 2010; Ihn et al., 2018). A large nationwide prospective study on AEs related to endovascular treatment in unselected patients with aneurysmal SAH has not been published before. In this study, we used a prospective nationwide cohort database of patients with non-traumatic SAH admitted to the neurosurgical centers in Sweden during a 3.5-year period (2014–2018). The aim of the study was to identify AEs related to the endovascular procedure, identify possible risk factors for the occurrence of AEs and to analyze their potential impact on functional outcome. We have previously published a study on AEs related to microsurgical occlusion of ruptured aneurysms (Baldvinsdóttir et al., 2023, Baldvinsdóttir et al., 2023a).

## 3 Material and methods

### 3.1 Study population and data collection

Data were extracted from a prospective nationwide database with information from the six neurosurgical centers in Sweden treating patients with aneurysmal SAH. The centers, situated in Gothenburg, Linköping, Lund, Stockholm, Umeå and Uppsala, cover the entire Swedish population, approximately 10 million individuals. Patients admitted from September 2014 to March 2018 diagnosed with spontaneous SAH were included. Microsoft Access® (Microsoft, Redmond, WA) was used to register the anonymized patient data. Two to three persons at each center were responsible for the prospective registration of the data and the follow-up of the patients at about one year after the bleeding. A steering group with one representative from each neurosurgical center designed the study and convened regularly to ensure coherence to database definitions and study protocol. As the study cohort did not include patients who died before reaching hospital or were in a too severe state to be transferred to a neurosurgical center, we did not check with other databases for missing cases. There was no conflict of interest. The data was not shared with the industry.

### 3.2 Clinical and radiological variables

An endovascular AE was defined as a complication or event linked to the endovascular procedure that was unexpected and potentially harmful to the patients, regardless of whether the event was avoidable or not. AEs registered in the database were periprocedural thromboembolism, aneurysmal re-rupture, vessel dissection and puncture site hematoma.

The specific endovascular treatment was registered (coiling, stent-assisted coiling, insertion of a flow diverter or a WEB device, or parent artery occlusion).

Baseline clinical parameters were registered; age, sex, body mass index, pre-existing comorbidities such as previous stroke, coronary heart disease, hypertension, diabetes, use of alcohol, and cigarette smoking. Neurological status prior to the endovascular procedure graded according to the World Federation of Neurosurgical Societies (WFNS) (Report of World Federation of, 1988), pupillary response, and focal neurological deficits were noted. Preoperative radiological findings such as severity of the bleeding according to the Fisher scale (Fisher et al., 1980), hydrocephalus, cerebral edema, and cerebral infarction and angiographic features of the aneurysm such as location, type (saccular, blister or other such as dissecting, fusiform or mycotic), size (maximal diameter) and neck configuration were registered. A wide neck was defined as neck size ≥ 4 mm and/or dome-to-neck ratio < 2 (Hendricks et al., 2019). Information concerning tranexamic acid infusion prior to endovascular treatment was registered. A post-treatment CT scan was performed within the first days following the endovascular treatment. We also recorded whether the patients developed delayed ischaemic neurological deficits (DIND) (Al-Tamimi et al., 2010) and if decompressive craniectomy was performed due to severe brain edema. All clinical variables were defined by the steering group prior to initiation of the study.

### 3.3 Outcome

Functional outcome was estimated using the extended Glasgow outcome scale (GOSE) (Jennett and Bond, 1975) one year after the bleeding as a standardized scale of neurological outcome including information regarding global disability and daily functions. The assessments were performed using a standardized questionnaire (Wilson et al., 1998) during an outpatient visit or telephone interview. The outcome scores were dichotomised into unfavorable outcome (GOSE 1–4, i.e., dead, or dependent on assistance of others) or favorable outcome (GOSE 5–8, i.e., able to live independently).

### 3.4 Statistical analysis

The impact of potential risk factors on AEs and the effects of AEs on functional outcome were analyzed using univariate and multivariate binary logistic regression. The variables which were statistically significant in the univariate analysis and variables of particular clinical relevance were included in the multivariate analysis. The number of variables included in each multivariate logistic regression were limited to one per every ten patients. A probability value of <0.05 was considered statistically significant. Odds ratio (OR) and 95% confidence interval (CI) were calculated through logistic regression. IBM SPSS® Statistics version 28 (IBM Corp., Armonk, NY) was used for the statistical analyses.

### 3.5 Ethics

The Regional Ethical Review Board in Stockholm granted permission to conduct the study (2014/990–3). Informed consent to participate was obtained either from the patients or their next of kin.

## 4 Results

### 4.1 Clinical characteristics

In total, 1037 patients were treated for a ruptured aneurysm during the study period. Endovascular occlusion was performed in 715 patients (69%). Microsurgery was performed in 322 patients (31%). Baseline clinical characteristics for the patients receiving endovascular treatment are shown in Table 1. The median age was 59 years. 488 (68%) of the patients were women. Prior to aneurysm occlusion, 422 (60%) were WFNS grade I-III and 282 (40%) WFNS grade IV-V. 91 patients (13%) had a focal neurological deficit before treatment. The most common comorbidities or associated lifestyle factors were cigarette smoking (392 patients, 55%), hypertension (284 patients, 40%) and alcohol use (100 patients, 14%). During the following clinical course, 179 patients (25%) developed DIND. Eleven patients (1.5%) were operated with decompressive craniectomy due to severe cerebral edema.

**Table 1:** Baseline clinical characteristics.

	n = 715
Age (median, range)	59 years (6–91)
Female sex	488 (68%)
Male	227 (32%)
WFNS grade	n = 704
I – III	422 (60%)
IV – V	282 (40%)
Focal neurological deficits	91/687 (13%)
Pathological pupillary light reflexes	32/707 (4.5%)
Pre-existing comorbidities	n = 715
Cigarette smoking	392 (55%)
Hypertension	284 (40%)
Alcohol use	100 (14%)
Coronary heart disease	65 (9.1%)
Previous stroke	35 (4.9%)
Diabetes	33 (4.6%)

WFNS, World Federation of Neurosurgical Societies.

### 4.2 Radiological characteristics

Table 2 shows baseline radiological characteristics. Fisher grade 4 SAH was the most common radiological finding, seen in 321 patients (46%). Half of the patients, 355 (50%) had hydrocephalus on the initial CT. Infarction and edema were less common (5% and 11%, respectively). The most common aneurysm location was on the anterior cerebral artery/anterior communicating artery (ACA/ACoA), 296 patients, 41%. The aneurysm was on the internal carotid artery (ICA) in 207 patients (29%) and in the posterior circulation in 165 patients (23%). In 47 patients (6.6%) the aneurysm was located on the middle cerebral artery (MCA). Most aneurysms (589, 86%) had a diameter of 10 mm or less. 244 (36%) were smaller than 5 mm. A total of 320 (52%) of the aneurysms had a wide neck. Most aneurysms were saccular (515, 72%) while 25 (3.5%) were blister aneurysms and 175 (24%) were of other types (dissecting, fusiform or mycotic).

**Table 2:** Baseline radiological characteristics.

Fisher grade (n = 700)	
1	23 (3.3%)
2	145 (21%)
3	211 (30%)
4	321 (46%)
Hydrocephalus	355 (50%)
Infarction	36 (5%)
Edema	82 (11%)
Aneurysm location (n = 715)	
ACA/ACoA	296 (41%)
ACA-Proximal to ACoA	9
ACoA	260
ACA-Distal to ACoA	27
ICA	207 (29%)
ICA-Posterior communicating	127
ICA-Anterior choroidal	20
ICA-Ophthalmic	33
ICA-Bifurcation	13
ICA-Unspecified	14
MCA	47 (6.6%)
Posterior circulation	165 (23%)
VA/BA	84
PICA	37
SCA	21
PCA	21
AICA	2
Largest aneurysm dome diameter (n = 684)	
<5 mm	244 (36%)
5–10 mm	345 (50%)
>10 mm	95 (14%)
Aneurysm neck size (n = 616)	
0–3.9 mm	390 (63%)
≥ 4 mm	226 (37%)
Maximal dome -to-neck size ratio ≥ 2	424 (69%)
Maximal dome -to-neck size ratio < 2	192 (31%)
Neck size ≥ 4 mm and/or dome-to-neck ratio < 2	320 (52%)

ACA, anterior cerebral artery; ACoA, anterior communicating artery; ICA, internal carotid artery; MCA, middle cerebral artery; VA, vertebral artery; BA, basilar artery; PICA, posterior inferior communicating artery; SCA, superior cerebellar artery; PCA, posterior cerebral artery; AICA, anterior inferior communicating artery.

### 4.3 Endovascular treatment

The patients were treated with endovascular occlusion of the aneurysm at a median time of 18 h after admission to a neurosurgical center. Altogether, 551 patients (77%) were treated within 72 h after the documented time of rupture. Coiling was the most common treatment modality and was performed in 597 patients (83%), followed by stent-assisted coiling in 67 patients (9.4%); a stent or a flow diverter was used in 37 patients (5.2%). Parent artery occlusion was performed in 13 patients (1.8%). A WEB device was used in one patient. Seven patients (1.0%) were operated with evacuation of an intracerebral hematoma, either prior to (n = 3) or after (n = 4) the endovascular treatment. 311 patients (43%) had received tranexamic acid prior to endovascular occlusion of the aneurysm.

### 4.4 Endovascular AEs

Among the 715 treated patients, there were in total 115 treatment-related AEs due to the endovascular occlusion in 113 patients (16%), Table 3.

i) *Thromboembolism.* Reduced blood flow in a vessel during the endovascular treatment because of thromboembolism was seen in 78 patients (11%). Of these, 31 patients were treated with thrombolysis, 5 patients were treated with a stent or a balloon, one patient received a heparin bolus and another patient received intra-arterial injection with nimodipine. Thromboembolic events leading to vessel occlusion and cerebral infarction were seen in 37 patients (5.2% of all treated), 27 of which (3.8%) had worsening of neurological symptoms. Logistic regression analysis for risk factors related to thromboembolism is shown in Table 4. Factors that were significantly related to an increased risk of a thromboembolic event in the univariate analysis were administration of tranexamic acid prior to the endovascular treatment (OR 3.09, 95% CI 1.86–5.14, p < 0.01), edema on the initial CT (OR 2.03, 95% CI 1.09–3.76, p = 0.03), other endovascular method than coiling (OR 1.87, 95% CI 1.08–3.25, p = 0.03), and regular use of alcohol (OR 2.03, 95% CI 1.14–3.61, p = 0.02). In the multivariate analysis, aneurysm location on MCA (OR 3.11, 95% CI 1.32–7.34, p = 0.01), tranexamic acid prior to endovascular treatment (OR 3.00, 95% CI 1.74–5.18, p < 0.01), cerebral edema (OR 3.07, 95% CI 1.51–6.24, p < 0.01), endovascular treatment with other method than simple coiling (OR 1.99, 95% CI 1.08–3.68, p = 0.03), and alcohol use (OR 1.99, 95% CI 1.06–3.73, p = 0.02) were statistically significant independent risk factors. Aneurysm diameter <5 mm was associated with a reduced risk of thromboembolic events in both univariate and multivariate logistic analysis (OR 4.8, 95% CI 0.27–0.85, p = 0.01).
ii) *Intraprocedural aneurysm re-rupture.* Rupture of the aneurysm during the endovascular treatment occurred in 28 patients (3.9%). Parent artery occlusion was used in 4 of these cases. Logistic regression analyses for risk factors related to aneurysm re-rupture during the endovascular treatment are shown in Table 5. Significant factors in the univariate analysis were blister type aneurysm (OR 9.59, 95% CI 3.49–26.4, p < 0.01), aneurysm diameter <5 mm (OR 3.43, 95% CI 1.56–7.54, p < 0.01), endovascular treatment methods other than coiling (OR 2.95, 95% CI 1.32–6.55, p < 0.01), and alcohol use (OR 3.69, 95% CI 1.65–8.24, p < 0.01). Multivariate logistical regression analysis indicated that blister type aneurysm (OR 6.34, 95% CI 1.82–22.1, p < 0.01) and aneurysm diameter <5 mm (OR 2.88, 95% CI 1.28–6.50, p = 0.01) were significant risk factors for intraprocedural re-rupture.
iii) *Vessel dissection.* Vessel dissection occurred during the endovascular procedure in four patients (0.6%). In three patients, the dissection was treated with a stent.
iv) *Puncture site hematoma.* Five patients (0.7%) had puncture site hematoma after the procedure, for which one patient needed acute surgery.

**Table 3:** Endovascular adverse events in the 715 treated patients.

Endovascular adverse events	
Thromboembolism	78 (11%)
Re-rupture	28 (3.9%)
Vessel dissection	4 (0.6%)
Puncture site hematoma	5 (0.7%)

**Table 4:** Logistic regression analysis for thromboembolic events (n = 78).

	Univariate		Multivariate	
	OR (95% CI)	*P* value	OR (95% CI)	*P* value
Age (older)	0.98 (0.97–0.99)	0.03	0.98 (0.96–0.99)	0.03
Gender (female)	0.81 (0.50–1.33)	0.41		
BMI (higher)	1.03 (0.98–1.08)	0.22		
Alcohol use	2.03 (1.14–3.61)	0.02	1.99 (1.06–3.73)	0.03
Cigarette smoking	1.14 (0.71–1.83)	0.59		
Hypertension	0.83 (0.51–1.36)	0.47		
Diabetes	1.13 (0.39–3.31)	0.82		
Coronary heart disease	1.35 (0.64–2.86)	0.43		
Initial clinical status, WFNS	0.79 (0.66–0.94)	0.01	0.68 (0.55–0.83)	<0.01
Received Tranexamic acid prior to endovascular treatment	3.09 (1.86–5.14)	<0.01	3.00 (1.74–5.18)	<0.01
Aneurysm location: ACA/ACoA	0.82 (0.51–1.33)	0.42		
Aneurysm location: ICA	0.71 (0.41–1.24)	0.23		
Aneurysm location: MCA	2.06 (0.95–4.43)	0.07	3.11 (1.32–7.34)	0.01
Aneurysm location: Posterior circulation	1.36 (0.80–2.30)	0.26		
Aneurysm diameter <5 mm	0.55 (0.32–0.94)	0.03	0.48 (0.27–0.85)	0.01
Aneurysm diameter 5–10 mm	1.31 (0.82–2.11)	0.26		
Aneurysm diameter >10 mm	1.57 (0.85–2.88)	0.15		
Wide neck aneurysm	0.83 (0.51–1.36)	0.47		
Higher Fisher grade on initial CT	1.05 (0.94–1.19)	0.39		
Infarction on initial CT	1.02 (0.35–2.97)	0.97		
Edema on initial CT	2.03 (1.09–3.76)	0.03	3.07 (1.51–6.24)	<0.01
Hydrocephalus on initial CT	0.76 (0.47–1.22)	0.26		
Aneurysm type: Saccular	1.57 (0.89–2.80)	0.12		
Aneurysm type: Blister	1.12 (0.33–3.83)	0.86		
Treatment: Coiling	0.53 (0.31–0.93)	0.03		
Treatment: Other than coiling	1.87 (1.08–3.25)	0.03	1.99 (1.08–3.68)	0.03

ACA, anterior cerebral artery; AcoA, anterior communicating artery; ICA, internal carotid artery; MCA, middle cerebral artery; CT, Computed tomography.

**Table 5:** Logistic regression analysis for intraprocedural aneurysm re-rupture (n = 28).

	Univariate		Multivariate	
	OR (95% CI)	*P* value	OR (95% CI)	*P* value
Age (older)	0.99 (0.97–1.02)	0.64		
Gender (female)	1.74 (0.70–4.35)	0.28		
BMI (higher)	1.04 (0.98–1.11)	0.22		
Alcohol use	3.69 (1.65–8.24)	<0.01		
Cigarette smoking	1.29 (0.59–2.79)	0.52		
Hypertension	0.60 (0.26–1.37)	0.22		
Diabetes	1.63 (0.37–7.17)	0.52		
Coronary heart disease	1.21 (0.36–4.12)	0.76		
Initial clinical status, WFNS	1.05 (0.82–1.34)	0.72		
Received Tranexamic acid prior to endovascular treatment	0.62 (0.28–1.40)	0.25		
Aneurysm location: ACA/ACoA	1.44 (0.67–3.06)	0.35		
Aneurysm location: ICA	0.81 (0.34–1.94)	0.64		
Aneurysm location: MCA	0.52 (0.07–3.88)	0.52		
Aneurysm location: Posterior circulation	0.91 (0.36–2.27)	0.83		
Aneurysm diameter <5 mm	3.43 (1.56–7.54)	<0.01	2.88 (1.28–6.50)	0.01
Aneurysm diameter 5–10 mm	0.45 (0.20–1.01)	0.053		
Aneurysm diameter >10 mm	0.22 (0.03–1.65)	0.14		
Wide neck aneurysm	0.85 (0.39–1.85)	0.69		
Higher Fisher grade on initial CT	1.05 (0.89–1.25)	0.55		
Infarction on initial CT	0.69 (0.09–5.23)	0.72		
Edema on initial CT	1.30 (0.44–3.85)	0.63		
Hydrocephalus on initial CT	1.37 (0.64–2.94)	0.42		
Aneurysm type: Saccular	0.97 (0.42–2.24)	0.94		
Aneurysm type: Blister	9.59 (3.49–26.4)	<0.01	6.34 (1.82–22.1)	<0.01
Treatment: Coiling	0.34 (0.15–0.76)	<0.01		
Treatment: Other than coiling	2.95 (1.32–6.55)	<0.01	1.47 (0.54–4.00)	0.45

ACA, anterior cerebral artery; ACoA, anterior communicating artery; ICA, internal carotid artery; MCA, middle cerebral artery; CT, Computed tomography.

Statistical analysis was not performed for vessel dissection or puncture site hematoma due to the limited number of patients.

### 4.5 Functional outcome

Extended Glasgow outcome scale (GOSE) was used to assess functional outcome. The follow-up was performed 13 months (median) after the bleeding. 92 patients were lost to follow-up. Therefore, 623 patients (87%) were available for GOSE assessments. 393 (63%) had favorable outcome (GOSE 5–8) and 230 patients (37%) had unfavorable outcome (GOSE 1–4). Table 6 shows logistical regression analysis for factors related to increased risk of unfavorable outcome. Several factors were significantly associated with unfavorable outcome in the univariate regression analysis: aneurysm re-rupture during the endovascular occlusion of the aneurysm (OR 3.19, 95% CI 1.39–7.34, p < 0.01), poor initial clinical status, WFNS grade (OR 1.90, 95% CI 1.68–2.16, p < 0.01), higher Fisher grade (OR 1.18, 95% CI 1.01–1.38, p = 0.04), infarction on the initial CT (OR 4.88, 95% CI 2.31–10.3, p < 0.01), edema on the initial CT (OR 4.13, 95% CI 2.43–7.01, p < 0.01), hydrocephalus on the initial CT (OR 3.94, 95% CI 2.78–5.59, p < 0.01), coronary heart disease (OR 3.62, 95% CI 2.07–6.32, p < 0.01), diabetes (OR 3.25, 95% CI 1.47–7.17, p < 0.01), hypertension (OR 2.04, 95% CI 1.46–2.84, p < 0.01), saccular type of aneurysm (OR 1.64, 95% CI 1.12–2.40, p = 0.01), and increased age (OR 1.05, 95% CI 1.03–1.06, p < 0.01).

**Table 6:** Logistic regression analysis, unfavorable outcome (n = 230).

	Univariate		Multivariate	
	OR (95% CI)	*P* value	OR (95% CI)	*P* value
Age (older)	1.05 (1.03–1.06)	<0.01	1.04 (1.02–1.06)	<0.01
Female sex	1.00 (0.70–1.43)	0.995		
BMI (higher)	1.02 (0.99–1.05)	0.24		
Alcohol use	1.32 (0.83–2.08)	0.24		
Cigarette smoking	1.26 (0.90–1.75)	0.18		
Hypertension	2.04 (1.46–2.84)	<0.01	1.30 (0.85–1.99)	0.23
Diabetes	3.25 (1.47–7.17)	<0.01	1.74 (0.56–5.45)	0.34
Coronary heart disease	3.62 (2.07–6.32)	<0.01	1.48 (0.70–3.11)	0.30
Initial WFNS grade (worse)	1.90 (1.68–2.16)	<0.01	1.72 (1.48–2.00)	<0.01
Received Tranexamic acid prior to endovascular treatment	1.38 (0.99–1.93)	0.056	1.08 (0.67–1.74)	0.76
Aneurysm location: ACA/ACoA	0.89 (0.64–1.24)	0.50		
Aneurysm location: ICA	0.75 (0.52–1.08)	0.12		
Aneurysm location: MCA	1.53 (0.82–2.86)	0.18		
Aneurysm location: Posterior circulation	1.38 (0.94–2.01)	0.10		
Aneurysm diameter <5 mm	0.85 (0.60–1.20)	0.35		
Aneurysm diameter 5–10 mm	1.20 (0.86–1.67)	0.29		
Aneurysm diameter >10 mm	0.95 (0.58–1.54)	0.82		
Wide neck aneurysm	0.98 (0.69–1.39)	0.92		
Fisher grade (higher)	1.18 (1.01–1.38)	0.04	1.20 (0.89–1.62)	0.23
Infarction on initial CT	4.88 (2.31–10.3)	<0.01	3.08 (1.24–7.65)	0.02
Edema on initial CT	4.13 (2.43–7.01)	<0.01	2.31 (1.19–4.49)	0.01
Hydrocephalus on initial CT	3.94 (2.78–5.59)	<0.01	2.36 (1.52–3.64)	<0.01
DIND	0.47 (0.14–1.55)	0.22		
Aneurysm type: Saccular	1.64 (1.12–2.40)	0.01	1.31 (0.80–2.17)	0.29
Aneurysm type: Blister	1.38 (0.54–3.55)	0.50		
Aneurysm type: Dissection/Pseudo-aneurysm	1.37 (0.72–2.59)	0.34		
Endovascular treatment: Coiling	0.84 (0.54–1.30)	0.43		
Endovascular treatment: Other than coiling	1.19 (0.77–1.85)	0.43		
Thromboembolic event	0.78 (0.44–1.35)	0.37		
Periprocedural aneurysm re-rupture	3.19 (1.39–7.34)	<0.01	6.92 (2.28–20.9)	<0.01

ACA, anterior cerebral artery; ACoA, anterior communicating artery; ICA, internal carotid artery; MCA, middle cerebral artery; CT, Computed tomography.

Factors significantly associated with increased risk of unfavorable outcome in the multivariate logistical analysis were aneurysm re-rupture during the endovascular occlusion of the aneurysm (OR 6.92, 95% CI 2.28–20.9, p < 0.01), infarction on the initial CT (OR 3.08, 95% CI 1.24–7.65, p < 0.02), edema on the initial CT (OR 2.31, 95% CI 1.19–4.49, p = 0.01), hydrocephalus on the initial CT (OR 2.36, 95% CI 1.52–3.64, p < 0.01), worse initial clinical status, WFNS (OR 1.72, 95% CI 1.48–2.00, p < 0.01), and increased age (OR 1.04, 95% CI 1.02–1.06, p < 0.01).

## 5 Discussion

For several years, endovascular occlusion of ruptured intracerebral aneurysms has in many centers been the most common technique used to prevent rebleeding. In the present national patient material, endovascular occlusion was adopted in 69% of all treated aneurysms. The total rate of intraprocedural AEs during endovascular treatment was 16%, which agrees with previous studies (Pierot et al., 2010, 2020a).

The strength of the present study is the prospective collection of data for 3.5 years. Such a prospective, comprehensive, and nationwide analysis of AEs related to endovascular occlusion of ruptured intracerebral aneurysms has to our knowledge not been published before. Thus, the results reflect the total management of non-selected SAH patients treated with endovascular treatment. A more detailed comparison of the different types of endovascular treatment was not the aim of the study. A limitation of the study may be that the results are mainly relevant for patients treated in similar health care systems and neurosurgical centers with similar caseloads as in Sweden (catchment population of about 0.9–2.2 million in each center). A more extensive description of the cohort of the SAH patients studied is published elsewhere (Ronne-Engstrom et al., 2023).

The definition of a complication or AE is debatable. We have chosen a broad approach and included all events that were unwanted or unpredicted and potentially detrimental to the patient. Regardless of whether they caused an actual clinical worsening of the patient or not.

Thus, our results are more likely an overestimation than an underestimation of the risks of treatment. We believe that knowledge on all potential risks that may cause the patient harm is of importance in the clinical decision making, for example, in the choice between clipping and coiling of an aneurysm. We have recently published a similar study of AEs using the same database on SAH patients treated with microsurgical occlusion of the ruptured aneurysm (Baldvinsdóttir et al.).

### 5.1 Endovascular AEs

Thromboembolic events during the endovascular treatment were seen in 78 patients (11%). In the literature, this AE had been described in about 5–16% of cases (van Rooij et al., 2006; Pierot et al., 2020b; Nomura et al., 2018; Jun et al., 2016). Clot formation may occur on or within the catheter, on the coil material or within the parent artery, (van Rooij et al., 2006; Nomura et al., 2018) and was mostly treated with thrombolysis. Several factors were related to increased risk of thromboembolism: aneurysm location on the MCA, treatment with tranexamic acid, cerebral edema, endovascular method other than coiling, and alcohol use.

Endovascular treatment of an aneurysm located on the MCA has been linked to increased risk of thromboembolism in previous studies (Link et al., 2018; Choi et al., 2019; Kadkhodayan et al., 2015). MCA aneurysms often have a wide neck, are often anatomically complex and arise from parent arteries that are more distal and of a smaller caliber than other intracerebral aneurysms. Therefore, the treatments may take longer time and require more devices, which may lead to stasis and turbulence (non-linear flow). Consequently, MCA aneurysms are often more properly treated with microsurgery than with endovascular treatment. The administration of tranexamic acid prior to endovascular treatment of the aneurysm was related to three-fold increased risk (OR 3.0) of a thromboembolic event. This is in contradiction with results from the ULTRA study (Post et al., 2021) where there was not increased risk of thromboembolism when tranexamic acid was given before endovascular treatment. The causal relation of cerebral edema and thromboembolism could be that increased intracranial pressure may affect vessel hemodynamics (Schmidt et al., 2018). Another explanation could be that patients with bleeding and cerebral edema may be in a more prothrombotic state (Larsen et al., 2010; Kocur et al., 2020). Patients with a thromboembolic event did not have an increased risk of unfavorable functional outcome. The increased risk related to aneurysmal treatment with endovascular methods other than coiling is expected as larger and/or more complex aneurysms might be associated with the catheters and more material introduced into the vessels for an extended time (Kameda-Smith et al., 2018). Conversely, aneurysm size <5 mm was associated with a lower risk of thromboembolism. This is in line with findings in the CLARITY study (Pierot et al., 2010) which showed that thromboembolism was significantly more often seen in aneurysms larger than 10 mm.

Vessel occlusion causing new cerebral infarction was seen in 5.2% of treated patients. The infarctions were noted on CT in the days following treatment. The detection of infarctions might have been higher if Magnetic Resonance Imaging (MRI) had been used. However, such infarctions are nevertheless often clinically silent (Bond et al., 2017; Iosif et al., 2018). In fact, only 3.8% of all treated patients had a clinical neurological worsening following thromboembolic event during the endovascular procedure.

Intraprocedural aneurysmal re-rupture was seen in 28 patients (3.9%). Our results showed that blister aneurysm, aneurysm diameter <5 mm, and endovascular method other than coiling increased the risk of intraprocedural re-rupture. We saw a six-fold increased risk (OR 6.3) of rupture if the aneurysm was of blister type. The treatment of blister type aneurysms is known to be very challenging (Nasra et al., 2021). The three-fold (OR 2.9) increased risk of re-rupture in aneurysms smaller than 5 mm was also found in the ARETA study (Pierot et al., 2020a) and may be explained by the limited maneuver space and catheterization angles found within small aneurysms.

Vessel dissection was rare, occurring in only four patients and had no clinical consequences. Hematoma at the puncture site (commonly the femoral artery) was also rare, seen in five patients. It is our clinical experience that these hematomas are often noted but seldomly cause significant problems. In our database we only registered hematomas that demanded special treatment; only one patient required surgical treatment.

### 5.2 Impact of AEs on patient outcome

The majority (63%) of the patients had favorable one-year outcome. This is comparable to other studies on patients treated for ruptured aneurysms with endovascular treatment (Langham et al., 2009; Roquer et al., 2020; Kim et al., 2020) as well with our previously published study of patients treated with microsurgery (Baldvinsdóttir et al.). The only AE that had significant impact on clinical outcome after one year was intraprocedural aneurysm re-rupture with almost seven-fold increased risk of unfavorable outcome. These findings underscore the importance of avoiding intraprocedural aneurysm re-rupture, the risk of which was higher in very small aneurysms and in blister-type aneurysms.

## 6 Conclusion

Endovascular treatment is often the preferred treatment for occluding ruptured aneurysms in Sweden. Treatment-related AEs occurred in 16% of patients. Thromboembolism and intraoperative re-rupture were the most common AEs. Aneurysmal re-rupture was the AE that affected functional outcome of the patients one year after the bleeding.

## Declaration of competing interest

The authors declare that they have no known competing financial interests or personal relationships that could have appeared to influence the work reported in this paper.
